# Basophils in Skin‐Mediated Sensitization Drive Subsequent Lung Inflammation in Airway‐Challenged Mice

**DOI:** 10.1111/all.70093

**Published:** 2025-10-11

**Authors:** E Da Choi, David Voehringer, Daniel Radtke

**Affiliations:** ^1^ Department of Infection Biology University Hospital Erlangen and Friedrich‐Alexander University Erlangen‐Nuremberg (FAU) Erlangen Germany; ^2^ FAU Profile Center Immunomedicine (FAU I‐MED) Erlangen Germany

**Keywords:** anaphylaxis, basophil, IgE, lung, sensitization

## Abstract

**Background:**

Atopic dermatitis (AD) and asthma are observed as epidemiologically linked allergic comorbidities, both characterized by elevated systemic IgE levels and type 2 immunity. Basophils play a pivotal role in these responses by producing interleukin‐4 (IL‐4), which is essential for IgE synthesis and allergic inflammation. However, their specific impact on the progression from AD to asthma remains unclear.

**Methods:**

We utilized an AD model in basophil‐deficient Mcpt8Cre mice and temporarily basophil‐depleted mice, where topical application of the vitamin D analog MC903 induced the alarmin TSLP, resulting in AD‐like symptoms. In addition, we topically applied ovalbumin (OVA) as a model allergen to trace the allergen‐specific immune response. We determined allergen‐specific antibody formation by analysis of the germinal center reaction and measured serum antibody concentrations and IgE loading of basophils in the spleen and lung. We further challenged mice sensitized via the skin in anaphylaxis and allergic lung inflammation models.

**Results:**

Our results demonstrate that basophils promote loss of skin barrier integrity, allergen‐specific IgE formation, and subsequent allergic responses. Basophil depletion selectively during sensitization significantly reduced IgE‐dependent anaphylaxis and lung inflammation. In challenged lungs, reduced inflammation and eosinophilia were accompanied by lower levels of chemokines CCL17 and CCL24, which attract Th2 cells and eosinophils, respectively. Notably, *Il4* and *Il13* were not affected by basophil depletion during sensitization but were reduced in mice that permanently lack basophils.

**Conclusion:**

We find that basophils promote IgE formation and lung sensitization to skin‐encountered allergens and drive secondary allergen‐induced lung inflammation. We separate the role of basophils in sensitization from their effector function during anaphylaxis or lung inflammation relevant to envision novel strategies to prevent the development of allergic comorbidities.

## Graphical abstract

In an atopic dermatitis model, basophils promote inflammation, barrier disruption, and IgE production to skin‐encountered OVA. Basophils drive allergic sensitization via the skin, predisposing lungs to inflammation upon airway challenge with the same allergen. Basophil depletion during sensitization reduces lung eosinophilia and CCL17/CCL24 level, while additional basophil absence during challenge is required to also impair *Il4*/*Il13* expression.

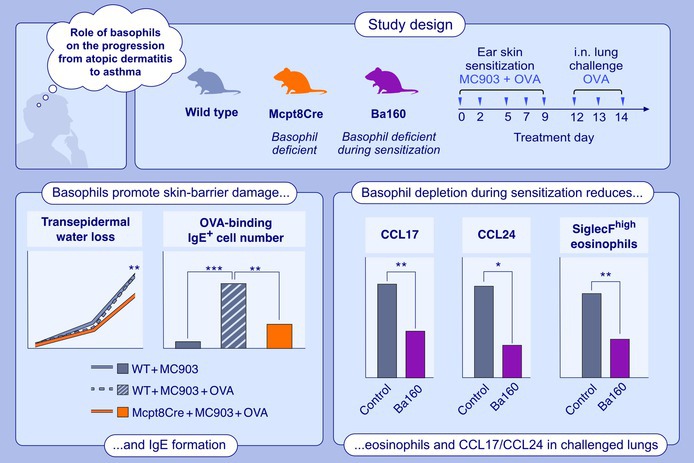

**Abbreviations**
CCL
: C‐C motif chemokine ligand
IgE
: immunoglobulin E
MC903
: calcipotriol
Mcpt8: mast cell protease 8
OVA
: ovalbumin
WT
: wild type

## 1 Introduction

Allergic diseases such as atopic dermatitis (AD) and asthma impose a significant health burden globally, affecting millions of individuals and highlighting the urgent need for a deeper understanding of their underlying mechanisms [1, 2, 3]. While an epidemiological link between AD and asthma is well‐established [4, 5, 6, 7, 8, 9] and can be replicated in mouse models [10, 11], the specific mechanisms driving this association remain poorly understood. Particularly, the role of immune cells in propagating allergic responses from the skin to the lung remains elusive. Among the various cells and pathomechanisms involved in allergic reactions, including damage/modulation of epithelium, release of alarmins, antigen presentation, activation of innate as well as adaptive immune cells, and IgE formation with a growing understanding of its diverse effects [12, 13], basophils have emerged as key players that are increased in AD skin [14]. They are known for their ability to produce critical amounts of interleukin‐4 (IL‐4), which is central to type 2 immune responses associated with allergies. They further promote loss of skin barrier integrity in different models [15, 16], thought to be associated with enhanced allergen uptake. A recent study in mice has demonstrated the essential role of basophils in systemic allergen sensitization via mechanically disrupted skin [17], yet it remains unclear if this holds true in an AD context and whether this is sufficient to induce or influence atopic comorbidities. To address this gap, our study employs a modified version of an established AD model [18] where we additionally apply allergen to the skin. As atopic diseases, AD and allergic asthma are defined by increased systemic IgE levels linked to adaptive immunity. The absence of adaptive immunity (including IgE) prevented mice with a genetic skin barrier defect from developing secondary spontaneous lung inflammation [11]. Therefore, we analyzed the impact of basophils on IgE formation and distribution as a likely driver of comorbidity. Our focus in this study was on elucidating how basophils drive lung inflammation through skin sensitization, providing novel insights into the progression from AD to asthma. By employing a modified AD model in combination with basophil‐deficient (Mcpt8Cre) [19] or temporarily basophil‐depleted mice, we aim to advance our understanding of the mechanistic link between AD and asthma to potentially inform new therapeutic strategies.

## 2 Results

### 2.1 Basophils Drive Local Skin Inflammation and Promote the Formation of Allergen Specific B Cells to Skin‐Applied Allergen

**FIGURE 1:**
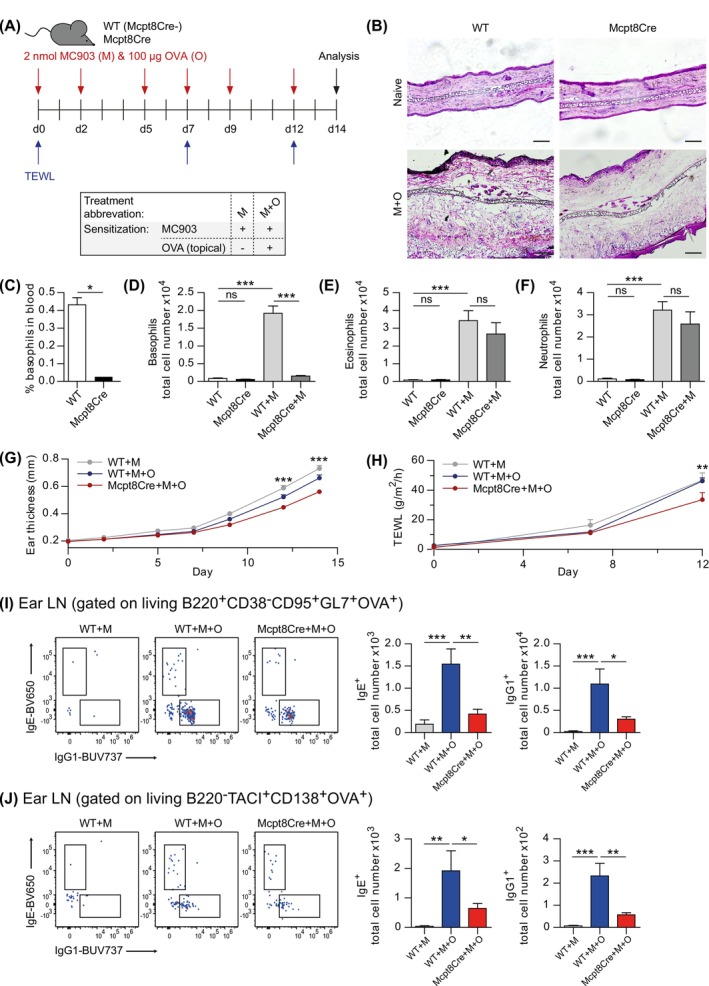
Basophils drive local sensitization via the skin. (A) Application scheme of MC903 and OVA to induce AD‐like symptoms and sensitization of WT and Mcpt8Cre mice via the ear skin. (B) Representative H&E ear staining, scale bar = 100 μm. (C) Quantification of basophils in blood. Quantification of (D) basophils, (E) eosinophils, and (F) neutrophils in ear skin. (G) Ear thickness and (H) TEWL over time. (I–J) Flow cytometric representation and quantification of OVA‐specific IgE^+^ and IgG1^+^ total GC B cells (I), or PCs (J). Data in (B) are one representative of three independent experiments and (C–F) are pooled data from two independent experiments with 7–13 mice per group. (G–J) are three independent pooled experiments with 6–7 mice per group. Bars and error bars represent mean + SEM. *p < 0.05; **p < 0.01; ***p < 0.001. Student's t‐test (C), one‐way ANOVA (D–F, I–J) or two‐way ANOVA (G–H).

To study the role of basophils in allergen sensitization via the skin, we applied an AD model that is similar to a recently published protocol [20]. In this, repeated topical application of the vitamin D3 analog MC903 (calcipotriol) to the ear induces the AD‐driving alarmin TSLP [18, 21], also found to be elevated in AD patients. TSLP is highly relevant in the MC903‐driven model, as TSLP‐deficient mice only develop mild disease symptoms [21]. Additionally to MC903, we applied ovalbumin (OVA), which serves as a traceable model‐allergen (Figure 1A), to assess the potency of sensitization via the skin against an otherwise harmless substance. In contrast to another model used to analyze lung sensitization in a skin inflammation context, we only applied the allergen on the inflamed skin for sensitization [10]. Furthermore, our model does not acutely mechanically disrupt the barrier, unlike tape‐stripping‐based models, which likely facilitate rapid allergen uptake before allowing time for the development of an AD‐like environment that we aim to analyze [17]. MC903‐induced TSLP acts on several cell types to promote AD symptoms, including activation of dendritic cells that promote Th2 polarization of CD4^+^ T cells, induction of basophil development, and ILC2 activation [22]. As basic pathology readouts for the AD setting, we performed histology, measured transepidermal water loss (TEWL; indicator of barrier integrity), determined ear swelling (inflammation), and performed flow cytometry for skin infiltrating effector cells. To analyze the role of basophils, we made use of basophil‐deficient (Mcpt8Cre) mice that also carry a fluorescent IL‐4 reporter (4get) [19]. In the AD model, Mcpt8Cre mice show slightly reduced pathology in H&E stained ear sections (Figure 1B) and while basophils are expectedly absent in blood and inflamed skin (Figure 1C–F), there is only a tendency for reduced infiltration of eosinophils and neutrophils to the ear compared to wild type (WT) (Figure 1E,F; gating strategy: Figure S1A). In line with previous findings, we observed reduced ear swelling and barrier damage in Mcpt8Cre mice [16, 23] (Figure 1G,H), suggesting reduced crossing of skin‐applied allergen through the more intact barrier. Next, we determined the local formation of B cells and antibody‐producing plasma cells (PCs) specific for skin‐applied OVA in the auricular and superficial cervical ear‐draining lymph nodes (ear LNs) by flow cytometry (Gating strategy: Figure S1B). The generation of high‐affinity antibodies, including allergy‐driving IgE, is dependent on B cell affinity maturation in germinal centers (GC) found in secondary lymphoid organs like LNs, which we analyzed. As expected, we hardly detected basophils in the LNs of Mcpt8Cre mice, while the number of PCs, CD4^+^ T cells, Th2 cells, or total IgG1^+^ and IgE^+^ GC B cells was similar between MC903 + OVA‐treated Mcpt8Cre mice and WT littermates (Figure S2A–I). In contrast, we observed a significant decrease in the amount of OVA‐specific IgG1^+^ and IgE^+^ B cells and PCs in the MC903 + OVA‐treated Mcpt8Cre mice (Figure 1I,J). As such, the general type 2 immune response in the LN seems not drastically altered. Therefore, the specific reduction in the response to skin‐applied allergen in basophil‐deficient mice rather implies that basophils promote barrier damage and inflammation that enhances allergen uptake via the skin to drive OVA‐specific immunity. Of note, we did not detect antibody‐specific GC B cells or PCs in the mediastinal, lung draining LN (Figure S2J,K), suggesting that OVA‐specific antibody formation is limited to the local skin‐draining LNs at the analyzed time point. The general development of AD‐like symptoms and sensitization via the skin was MC903‐dependent as treatment with the MC903 solvent ethanol (EtOH) alone or in combination with OVA did not induce AD‐like symptoms or sensitization (Figure S3). MC903‐dependent sensitization likely occurs by induction of type 2 immunity, which leads to downregulation of the epidermal differentiation complex in keratinocytes and hence makes the skin barrier more permeable to allergen. Taken together, these findings highlight that basophils are strong mediators of local inflammation and barrier damage that facilitate B cell and PC formation to skin‐applied allergen.

### 2.2 Systemic Sensitization for IgE‐Dependent Anaphylaxis Is Promoted by Basophils

**FIGURE 2:**
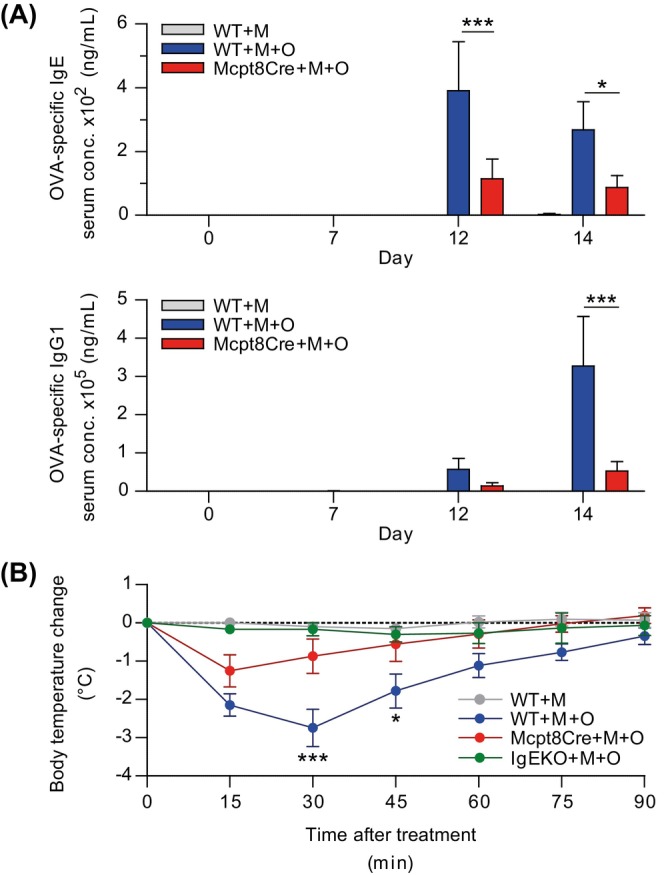
Basophils promote skin‐mediated sensitization for anaphylaxis. (A) Serum concentration of OVA‐specific IgE and IgG1 in mice sensitized via the ear skin over time. (B) Passive anaphylaxis experiment with serum transfer from indicated mice that were OVA‐sensitized via the ear skin into naive C57BL/6 recipients. Shown are body temperature changes of recipients over time upon i.v. Challenge with OVA. Data in (A–B) represents three independent pooled experiments with (A) 7–9 and (B) 9 mice per group except for the IgEKO group with 3 mice in total. WT = Mcpt8Cre‐ mice, Mcpt8Cre = basophil‐deficient mice, M = MC903, O = OVA. Bars and error bars represent mean + SEM. *p < 0.05; **p < 0.01; ***p < 0.001. Two‐way ANOVA (A–B).

Next, we analyzed how the locally formed antibody response is systemically distributed in the course of the MC903 model. In the serum, we find OVA‐specific antibodies with IgE first detected and peaking at day 12, and IgG1 rising from day 12 onwards with reduced levels in Mcpt8Cre mice compared to WT (Figure 2A). In contrast, the total IgE concentration was rising and IgG1 stayed unchanged until day 14, with similar concentrations measured across treatment groups (Figure S4). To understand how basophils modulate the systemic formation of humoral immunity to skin‐encountered allergen, we performed passive anaphylaxis experiments by transferring serum from sensitized WT or Mcpt8Cre mice into C57BL/6 mice that were subsequently challenged with 50 μg OVA i.v. In this system, basophils are present as effector cells for anaphylaxis in the recipient mice, and observed changes solely depend on the quantity and quality of the humoral immunity transferred with the serum. To test if the induced anaphylaxis is IgE dependent, we also added a group into which we transferred serum from sensitized IgE‐deficient (IgEKO) mice. Readout was the body temperature drop observed during the anaphylaxis reaction. Serum transfer from sensitized Mcpt8Cre mice led to a significantly milder drop in body temperature compared to serum from sensitized WT littermates, while serum transfer from only MC903 treated WT or OVA‐sensitized IgEKO mice did not induce changes in body temperature upon challenge (Figure 2B). This indicates that the humoral sensitization promoted by basophils is relevant to accelerate IgE‐mediated anaphylaxis.

### 2.3 Basophils in Sensitization via the Skin Pre‐Dispose for an Elevated Airway Challenge Response

**FIGURE 3:**
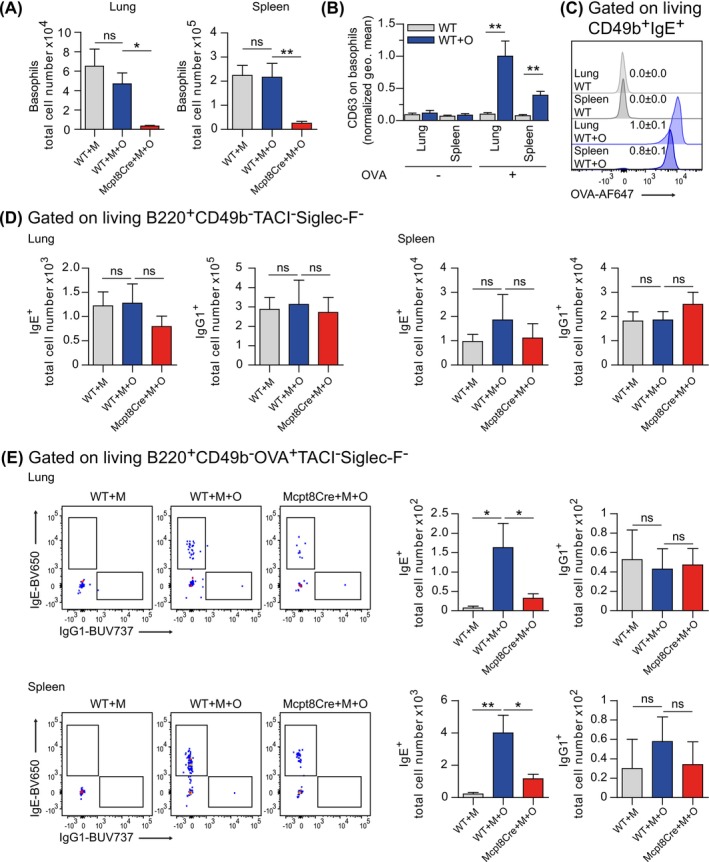
Allergen‐sensitization of distal organs via the skin is enhanced by basophils. (A) Quantification of basophils in lung and spleen. (B) Ex vivo restimulation of lung and splenic basophils from OVA‐sensitized mice. Expression of the activation/degranulation marker CD63 on basophil surfaces is shown. (C) Flow cytometric MFI analysis of fluorescent OVA bound to basophils. (D) Quantification of IgE^+^ and IgG1^+^ total B cells in lung and spleen. (E) Flow cytometric analysis and quantification of OVA‐specific IgE^+^ and IgG1^+^ total B cells in lung and spleen. Data in (A, D–E) represent four independent pooled experiments with 8 mice per group. Data in (B) represents three independent pooled experiments with 6–7 mice per group. WT = Mcpt8Cre‐ mice, Mcpt8Cre = basophil‐deficient mice, M = MC903, O = OVA. Bars and error bars represent mean + SEM. *p < 0.05; **p < 0.01; ***p < 0.001. One‐way ANOVA (A–E).

**FIGURE 4:**
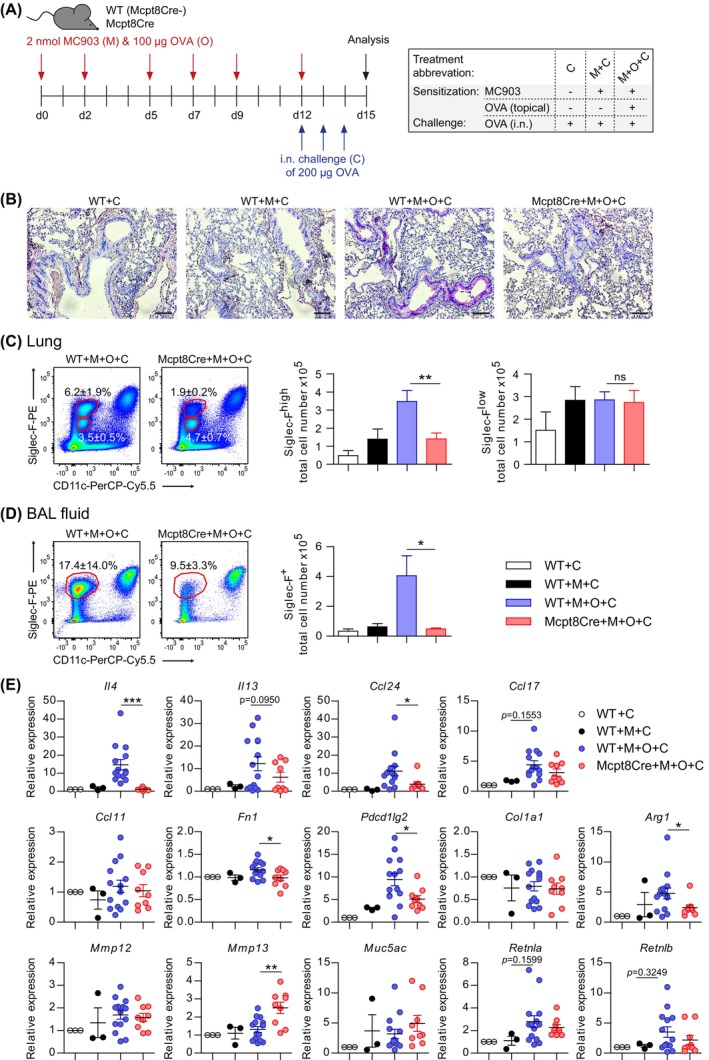
Basophils in sensitization via the skin pre‐dispose for an elevated airway challenge response. (A) Application scheme to promote AD‐like symptoms, sensitization and subsequent lung challenge. (B) Representative H&E lung staining, scale bar = 100 μm. (C) Flow cytometric representation of Siglec‐F^high^ and Siglec‐F^low^ total lung tissue living cells with quantification. (D) Flow cytometric representation of Siglec‐F^+^ total BAL fluid living cells with quantification. (E) Relative expression (qRT‐PCR) of different marker genes in challenged lung tissue. Data in (B) are from one representative out of three independent experiments and (C, E) are three independent pooled experiments with 9–14 mice per group except WT and WT + M group with 3 mice. Data in (D) represents 5 mice per group. Bars and error bars represent mean + SEM. *p < 0.05; **p < 0.01; ***p < 0.001. One‐way ANOVA (C–E).

After discovering that basophils promote the serum antibody response specific for skin‐encountered allergen, we next analyzed if cells in the lung are also sensitized by allergen application to the skin. To analyze skin‐mediated sensitization, we determined loading of lung basophils with OVA‐specific IgE upon sensitization via the skin. As Mcpt8Cre mice lack basophils, we only determined basophil sensitization in WT mice (Figure 3A). There, we find that basophils of OVA‐treated mice were loaded with OVA‐specific antibodies. As a reference, we also determined the loading of basophils in the spleen. To assess the functional relevance of the bound OVA‐specific antibodies, we stimulated basophil‐containing single‐cell suspensions of lung and spleen from the WT and the WT + OVA group (day 14) with OVA ex vivo. We measured enhanced CD63 expression, which is a readout for activation/degranulation on basophils from lung and spleen of the WT + OVA group, but not the WT group. Compared to spleen, lung basophils of the WT + OVA group showed enhanced upregulation of the activation/degranulation marker CD63 on the surface (Figure 3B). We further assessed the mean fluorescence intensity (MFI) of labeled OVA bound to the surface of basophils and observed that basophils in the lung of the WT + OVA group exhibited a higher MFI of bound OVA as compared to basophils in the spleen (Figure 3C). While cells of both organs are sensitized, it indicates higher inflammation potential of sensitized lung basophils compared to basophils from spleen. This highlights efficient sensitization of basophils in the lung. We also find OVA‐specific B cells in the spleen and the lung of mice sensitized via the skin. To enhance OVA‐specific B cell detection, we excluded non‐target events, including basophils (CD49b^+^), plasma cells (TACI^+^), and eosinophils (Siglec‐F^+^), while addressing potential autofluorescence through selection of negative events from these channels (Gating strategy: Figure S5). Although the total number of IgG1^+^ and IgE^+^ B cells and the number of OVA‐specific IgG1^+^ B cells are similar in lung and spleen of Mcpt8Cre and WT mice, the OVA‐specific IgE^+^ B cells are reduced in sensitized Mcpt8Cre mice (Figure 3D,E). We confirmed the specificity of the OVA‐specific IgE^+^ B cell stain by analysis of sensitized IgEKO mice, which expectedly lack OVA‐specific IgE^+^ B cells (Figure S6). To find out if the observed sensitization to skin‐encountered OVA is sufficient to excel allergic lung inflammation, we challenged WT and Mcpt8Cre mice at day 12 of the WT + OVA model by application of 200 μg OVA intranasally for three consecutive days. As controls, we included an untreated WT group and WT mice that were MC903 treated but without topical application of OVA (Figure 4A). Histologic analysis of lung tissue sections suggests stronger inflammation in sensitized WT as compared to Mcpt8Cre mice (Figure 4B). Then, we determined eosinophil numbers in total lung tissue and bronchoalveolar (BAL) fluid, which are a common readout in allergic lung inflammation. We observed a tendency for more inflammatory Siglec‐F^high^ eosinophils induced by the MC903 model alone. In the OVA‐sensitized groups, Mcpt8Cre mice recruited significantly less inflammatory Siglec‐F^high^ eosinophils as compared to WT controls. In contrast, we did not observe changes in the Siglec‐F^low^ eosinophil populations (Figure 4C,D). Transcriptionally, we find reduced levels of *Il4* in Mcpt8Cre mice indicating a reduced type 2 immune response and diminished upregulation of *Ccl24* which encodes for eotaxin‐2 and drives eosinophil recruitment. The eotaxin‐1 encoding gene *Ccl11* and Th2 cell recruiting *Ccl17* were not altered. *Fn1* (tissue repair, fibrosis), *Arg1* (immune modulation, tissue repair), and *Pdcd1lg2* (immune regulation/tolerance), which are upregulated in lung inflammation and asthma signatures [24, 25, 26, 27], showed significantly reduced expression on gene level in Mcpt8Cre mice compared to WT upon lung challenge, indicative of reduced inflammation in basophil‐deficient mice. In the challenge situation, we further find a higher level of *Mmp13* in Mcpt8Cre mice compared to WT, which encodes for an antifibrotic metalloproteinase and might indicate reduced lung damage in the absence of basophils. The markers *Col1a1*, *Muc5ac*, *Retnla*, and *Retnlb* are unchanged and indicate that basophils selectively modulate gene expression in our model (Figure 4E). These results suggest that basophil impact on sensitization to skin‐encountered allergen can be relevant to drive allergic lung inflammation.

### 2.4 Basophil Promotion of Skin‐Mediated Sensitization on Its Own Exacerbates Allergic Lung Inflammation Independent of Basophil Effector Functions in Lung Inflammation

**FIGURE 5:**
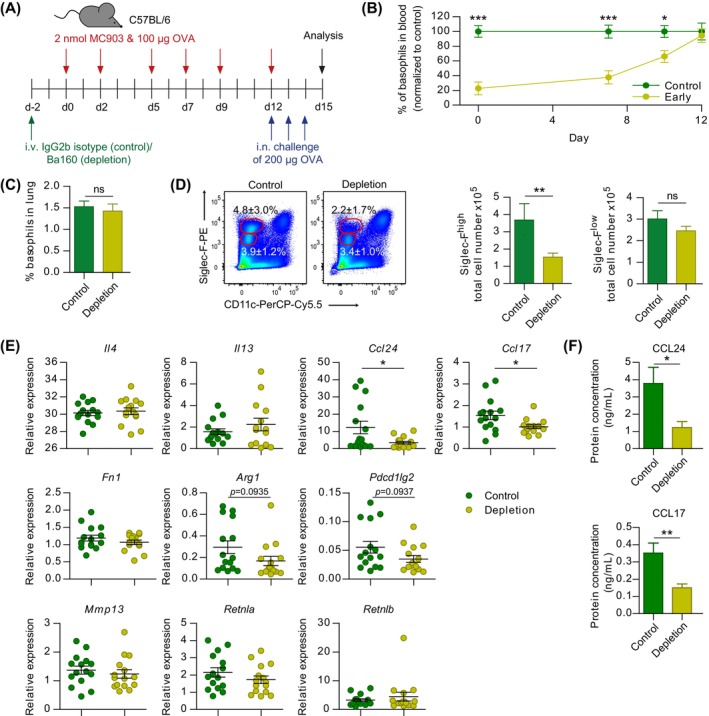
Basophils promote skin‐mediated sensitization of the lung independent of their presence during airway challenge. (A) Application scheme to induce AD‐like and lung challenge symptoms in C57BL/6 mice that were either temporally basophil‐depleted (anti‐CD200R3 clone Ba160 i.v.) named “depletion” group or injected with IgG2b isotype antibody named “control” group. (B) Quantification of basophils in blood over time in treatment groups. (C) Quantification of basophils in lung at day 15 of the lung challenge model. (D) Flow cytometric representation of Siglec‐F^high^ and Siglec‐F^low^ total lung tissue living cells with quantification. (E) Relative expression (qRT–PCR) of different marker genes in challenged lung tissue. (F) Protein concentration of CCL24 and CCL17. Data in (B–F) are from two independent pooled experiments with (B) 10–15 mice per group, (C–F) 15 mice per group. Bars and error bars represent mean + SEM. *p < 0.05; **p < 0.01; ***p < 0.001. Two‐way ANOVA (B) or Student's t‐test (C–F).

While our experiments with Mcpt8Cre mice provide evidence that basophils enhance sensitization to skin‐encountered allergen, it is still possible that the observed differences in subsequent allergic lung inflammation are caused by missing basophil effector functions during lung challenge. To dissect the role of basophils in skin‐mediated sensitization from their role in lung allergen challenge, we injected 50 μg anti‐CD200R3 antibody i.v. Into WT mice 2 days before we started the AD+OVA model to deplete basophils selectively in the sensitization phase [17, 28]. Control mice were injected with 50 μg of isotype control antibody (Figure 5A). In the depletion group, we observed about 80% reduction of basophils (CD49b^+^IgE^+^) in the blood at the start of OVA sensitization. Of note, murine mast cells are CD49b^−^ and are therefore excluded from the basophil gate. Basophil numbers recovered over time and were not significantly different in blood and lung between the basophil‐depletion and the control group when we started the lung challenge at day 12 (Figure 5B,C). Thus, basophils were available as effector cells in the lung‐challenge phase of the model. Nevertheless, we also observed a reduced recruitment of inflammatory Siglec‐F^high^ eosinophils in the lung of mice that were basophil‐depleted in the sensitization phase while Siglec‐F^low^ eosinophil populations were not altered (Figure 5D).

In contrast to the challenge experiment with Mcpt8Cre mice (Figure 4E), we find similar levels of *Il4* and *Il13* expression in challenged lungs when basophils are only missing in the sensitization phase compared to non‐depleted controls, indicative of a functional type 2 immune response in the effector phase. However, *Ccl24* remains reduced and hence seems dependent on basophils in the sensitization phase. *Ccl17*, which showed only a reduction tendency in the lung challenge experiment with Mcpt8Cre mice, is even significantly reduced in temporarily basophil‐depleted mice compared to controls. The expression of *Fn1*, *Arg1*, *Mmp13*, *Retnla*, and *Retnlb* genes is similar in temporarily basophil‐depleted mice compared to controls (Figure 5E). Thus, most differences and tendencies observed in the lung challenge of basophil‐deficient Mcpt8Cre mice are likely explained by basophil effector functions in the lung challenge phase. The reduction and significant difference of *Ccl24* and *Ccl17* is further confirmed on the protein level (Figure 5F). This data helps to untangle the role of basophils in skin‐mediated sensitization from effector functions in the lung. Taken together, basophil‐driven sensitization via the skin pre‐disposes the lung for an elevated airway challenge response.

## 3 Methods

### 3.1 Mice

Basophil‐deficient Mcpt8Cre mice [19], Mcpt8Cre‐negative (WT) littermates (which both also carry an IL‐4 IRES‐eGFP reporter) [29], IgE‐deficient (IgEKO) mice [30] and C57BL/6 mice (Charles River Laboratories, Wilmington, USA) were used. All mice were on a C57BL/6 background and 8–12 weeks of age. Animal experiments were approved by the Regierung von Unterfranken and performed in accordance with the German animal protection law.

### 3.2 Sensitization via the Skin and In Vivo Lung Challenge

For the AD model, both ears of mice were treated by topical application of 2 nmol MC903 (Cayman Chemical, Ann Arbor, USA) dissolved in 20 μL EtOH, and 10 μL were applied to each side of the ears. Treatment was repeated every second day until analysis at day 14, if not differently specified. In case of OVA sensitization, 100 μg ovalbumin (OVA) (Sigma‐Aldrich, St. Louis, USA) in 40 μL PBS was added per mouse when EtOH had evaporated after MC903 treatment (10 μL per side of the ear). For lung challenge experiments, mice were sensitized via the skin until day 12. Then, sensitized mice were intranasally (i.n.) challenged with 200 μg of OVA from day 12 onwards for three consecutive days and analyzed at day 15.

### 3.3 Ear Thickness and Transepidermal Water Loss (TEWL) Measurement

Ear thickness was measured at the upper side using an electronic caliper (INSIZE, Suzhou New District, China). Transepidermal water loss (TEWL) was assessed with a Tewameter TM Nano device (Courage + Khazaka Electronic GmbH, Koeln, Germany). TEWL was measured for at least 90 s until values stabilized.

### 3.4 Basophil Depletion

Basophils in C57BL/6 mice were depleted 2 days prior to sensitization through intravenous (i.v.) injection of 50 μg of anti‐CD200R3 antibody (clone Ba160, BioLegend, San Diego, USA). Control mice received 50 μg of isotype control antibody (clone RTK4530, BioLegend).

### 3.5 Passive Anaphylaxis

C57BL/6 mice were passively sensitized by i.v. Injection of 200 μL serum from ear‐sensitized WT or Mcpt8Cre mice. After 24 h, mice were challenged with 200 μg of OVA i.v. Body temperature was rectally measured at indicated time points after challenge.

### 3.6 Flow Cytometry and Tissue Preparation

Single‐cell suspensions were prepared from ear LNs, mediastinal LNs, spleens, and lungs (perfused with PBS) by mashing through cell strainers. Lungs were cut into pieces and digested with DNase DN25 (Sigma–Aldrich) and Liberase (Roche, Basel, Switzerland) at 100 μg/mL for 30 min at 37°C. ACK buffer (0.15 M NH_4_Cl, 1 mM KHO_3_, 0.1 mM Na_2_EDTA) was used for erythrocyte lysis in samples of spleen and lung. Before surface staining, the cells were treated using acetate buffer to remove cytophilic IgE as described [31]. Fc‐Block was used (clone 2.4G2) before staining with antibodies for 30 min at 4°C (antibody list and dilutions in Table S1). The cells were further fixed and permeabilized using Cytofix/Cytoperm (Becton Dickinson, BD, Franklin Lakes, USA) before IgE intracellular staining with Phosflow Perm/Wash Buffer (BD). Cells were analyzed using a BD LSRFortessa flow cytometer (BD) and FlowJo software (BD). To determine total cell numbers, we counted living cells (trypan blue negative) of single‐cell suspensions from different organs (for ear skin, the numbers reflect total cells of both ears combined). To calculate the total number per cell population, we determined the percentage of each population from the living cells (Fixable Viability Dye negative) by flow cytometry and multiplied it by the number of counted living cells.

### 3.7 ELISA and LEGENDplex Assay

OVA‐specific or total IgE and IgG1 serum levels were determined using standard ELISA methods. In short, anti‐IgE (clone R35‐72, BD), anti‐IgG1 (H + L) (SouthernBiotech, Birmingham, USA), or OVA were used for coating and 3% bovine serum albumin for blocking. Then serum samples or standards were added (IgE κ Isotype (BD) or IgG1‐unlabelled (SouthernBiotech)). Anti‐mouse IgE or anti‐mouse IgG1, both coupled to alkaline phosphatase (SouthernBiotech), were used as detection antibodies.

For bead‐based chemokine measurement, the middle lobe of the right lung side was homogenized with OMNI Bead Ruptor (Biolabproducts GmbH, Bebensee, Germany) (3 × 3.10 m/s each 15 s) in RIPA lysis buffer (1% NP‐40, 50 mM Tris pH 7.4, 0.15 M NaCl, 1 mM EDTA, 0.5% C_24_H_39_NaO_4_), and the resulting supernatant was analyzed with LEGENDplex Kits 741,068 and 740,451 (BioLegend).

### 3.8 RNA Extraction and qRT‐PCR Assay

Middle lobe of right lung side was homogenized and lysed in TRIzol Reagent (Invitrogen) for RNA isolation. Quantitative RT‐PCR was performed using SYBR Green on a ViiA 7 Real‐Time PCR System with TaqMan Array Block (Thermo Fisher, Waltham, USA). Primers are listed in the supplement (Table S2).

### 3.9 Histology

Cryosections were generated from ears (7 μm thick) and paraffin sections from lungs (3 μm thick). Sections were stained with hematoxylin and eosin and imaged with an Axio Vert. A1 microscope and processed with ZEN software (both Zeiss, Jena, Germany).

### 3.10 Statistical Analysis

GraphPad Prism 5 (Dotmatics, San Diego, USA) was used for statistical analysis. Student's t test was employed for comparisons between two groups, whereas a one‐way ANOVA followed by Bonferroni correction was used to assess comparisons between multiple groups. Alternatively, one‐way ANOVA with Dunnett's test was applied when each treatment group is compared specifically to a control group. When the experimental data includes a covariate such as time, a two‐way ANOVA with repeated measures and Bonferroni correction was used. Data is presented as mean + SEM.

## 4 Discussion

Granulocytes are important effector cells in allergy, and especially the context‐dependent role of basophils in different stages of allergic responses improved in the last years [17, 23, 32, 33, 34, 35]. This study demonstrates that basophils drive lung sensitization to skin‐applied allergen. The mechanism might be evolutionarily relevant in the defense against airborne pathogens that are encountered by the skin but are also likely to be inhaled and reach the lung. We and others observe that basophils promote barrier damage in the MC903 model [16], which likely facilitates barrier crossing of allergen. However, even in a model in which the barrier is mechanically impaired by tape stripping, likely enhancing allergen penetration independent of basophils, there is a reduced allergen presentation by dendritic cells and reduced systemic sensitization when basophils are missing. This was determined by measuring serum antibody concentrations specific to skin‐applied allergen and in vitro restimulation of splenocytes [17] and suggests basophil contribution to allergic sensitization beyond their role in barrier disruption. For example, basophils home to secondary lymphoid organs during type 2 immune responses and likely contribute to a type 2 bias of the GC and the subsequent humoral response [36]. These aspects are relevant not only for allergy but also for vaccine development against skin‐encountered parasites, as basophils contribute to protection against secondary *Nippostrongylus brasiliensis* infection in mice [37]. At the time points we analyzed, adaptive immunity to skin‐applied allergen formed rather locally in skin‐draining LNs. This differs from allergens that are systemically spread post‐barrier crossing, as seen with skin‐penetrating nematodes [38]. Thus, local treatment of the skin might be beneficial to reduce the risk of developing distant comorbidities. Nevertheless, OVA‐specific IgE distributes in our model, and binding of allergen‐specific IgE to Fc‐receptors on mast cells and basophils can induce antibody‐mediated systemic anaphylaxis upon allergen binding in mice [35, 39]. While such an AD‐dependent mechanism in humans is supported by empirical data [40, 41], mechanistic understanding is missing. The reduced allergen‐specific antibodies to skin‐encountered allergen in the serum of Mcpt8Cre mice suggest that basophils are relevant in systemic sensitization as a prerequisite for anaphylaxis. Further, the allergen‐specific antibody concentration in the serum might be an indicator for the sensitization of distal organs and development of AD‐related comorbidities like asthma. Interestingly, higher degranulation marker expression after challenge of basophils from lungs compared to spleens of OVA‐sensitized mice suggests differences in OVA‐specific antibody availability or organ‐specific activation thresholds. In addition to their role in sensitization, basophils are effector cells in IgE‐mediated anaphylaxis [33] and allergic lung inflammation [34, 42, 43] in mice, highlighting their relevance in different allergic contexts. Notably, basophil effector function in a lung house dust mite model was independent of IgE [34], suggesting that potential differences in IgE loading of repopulating basophils in our antibody‐mediated basophil depletion setup might not affect basophil function upon lung challenge. However, relevant basophil contribution to lung inflammation seems context‐dependent, and basophils were dispensable in other setups [19, 44, 45]. Basophils are also capable of mediating the recruitment of other type 2 effector cells [23, 42] and induce eosinophil recruitment in an urticarial model, likely by promotion of *CCL24* expression [23]. As we also find that basophils promote CCL24, this is probably a relevant factor for basophil‐mediated eosinophil recruitment in lung challenge of mice sensitized via the skin. This study uniquely separates the role of basophils in skin‐mediated sensitization from their downstream effector functions to understand how basophils promote predisposition to allergic multimorbidity. While focusing on anaphylaxis and lung inflammation as models for asthma development, the findings may also have implications for other allergic comorbidities, such as food allergy, which is associated with cutaneous inflammation in humans [40]. However, as any mouse model, the MC903 and OVA sensitization model we used can only partially recapitulate the situation in AD patients. It does not reflect different disease endotypes or heterogeneity in the human population. In addition, the structure and composition of the skin in mice and humans differ. Nevertheless, this model might help to unravel mechanisms that are conserved between mouse and human to envision strategies to prevent disease. Furthermore, in combination with other mouse models, like filaggrin mutant mice that have an impaired skin barrier and also show sensitization to skin‐applied allergen [46], shared and unique pathomechanisms might be discovered that can help to understand human disease endotypes. Based on our findings, drugs that limit basophil activity in the skin, like the phosphodiesterase 4 inhibitor Difamilast [47], might bear potential to interfere with skin‐mediated sensitization [47]. Future research should explore the potential of new and existing drugs used in the treatment of AD for their potential to prevent basophil‐driven comorbidities.

## Author Contributions

D.R. and D.V. conceptualized the study. E.D.C., D.R., and D.V. designed experiments; E.D.C. and D.R. performed experiments and analyzed the data. E.D.C., D.R., and D.V. reviewed the data, contributed to interpretation, and edited the manuscript. D.R. and D.V. acquired the funding for this study. All authors approved the final submitted version of the manuscript.

## Conflicts of Interest

The authors declare no conflicts of interest.

## Supporting information


**Figure S1.** Gating strategy for granulocyte subsets, GC B cells and PCs.

**Figure S2.** Quantification of basophils, CD4^+^ T, Th_2_, total IgE^+^ and IgG1^+^ GC B cells and PCs in ear LN; and OVA‐specific GC B cells and PCs in med LN.

**Figure S3.** Quantification and measurement of various control groups.

**Figure S4.** Concentration of total IgE and IgG1 in serum.

**Figure S5.** Gating strategy for OVA‐specific B cells.

**Figure S6.** Gating strategy and quantification of OVA‐specific IgE in WT and IgEKO mice.

**Table S1.** List of antibodies used for immunofluorescence staining.
**Table S2.** List of primers used for qRT‐PCR analysis.

## Data Availability

The data that support the findings of this study are available from the corresponding author upon reasonable request.
