# Supplementary figures and images for: Basophils in Skin‐Mediated Sensitization Drive Subsequent Lung Inflammation in Airway‐Challenged Mice
Source: Allergy. 2025 Oct 11;81(1):220–31. doi: 10.1111/all.70093 (PMC12773652; doi:10.1111/all.70093)

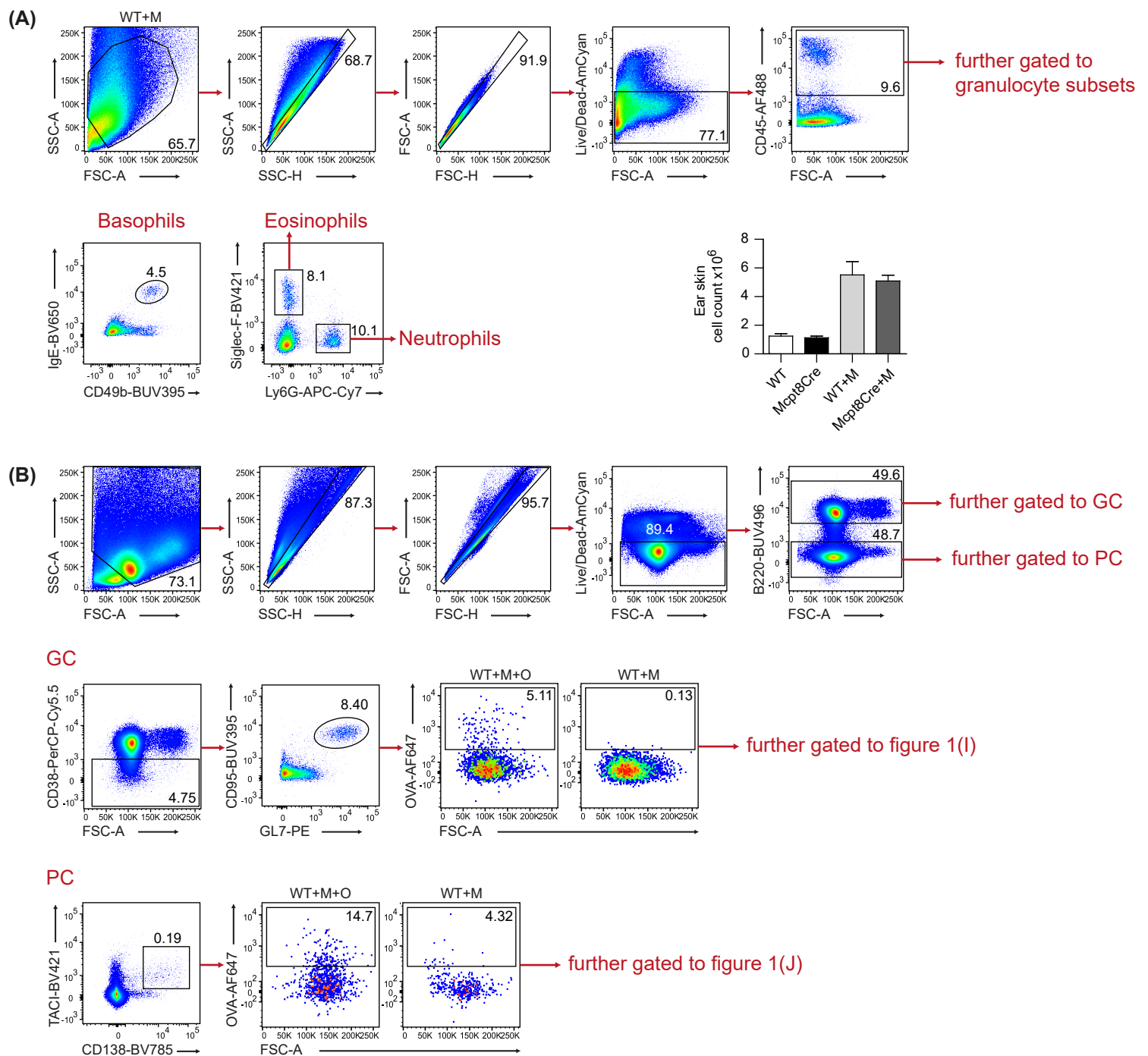

Supplementary figure 1\_Choi et al.

Supplement: Supplementary file 1 — Figure S1. Gating strategy for granulocyte subsets, GC B cells and PCs. [file ALL-81-220-s003.pdf]

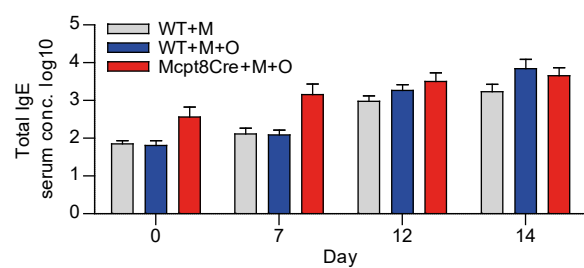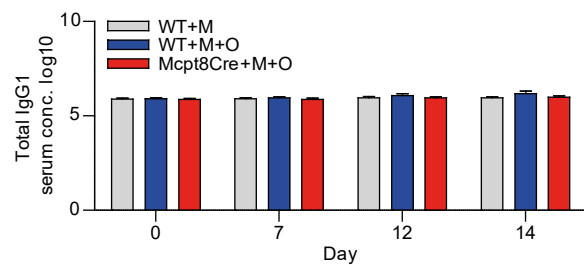

Supplementary figure 4\_Choi et al.

Supplement: Supplementary file 4 — Figure S4. Concentration of total IgE and IgG1 in serum. [file ALL-81-220-s004.pdf]

## Lung

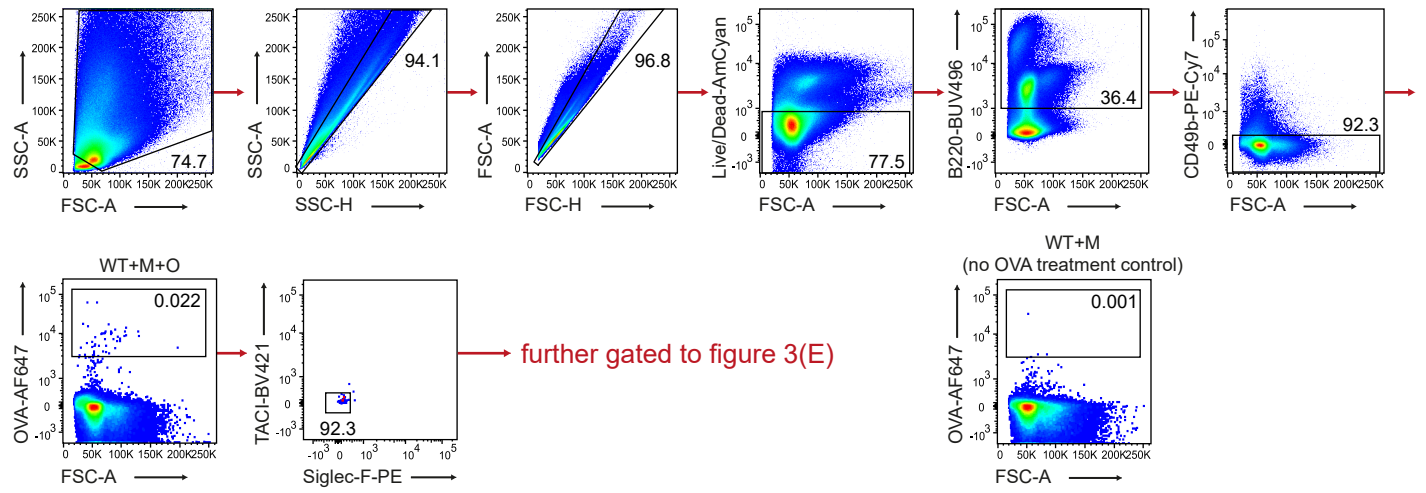

## Spleen

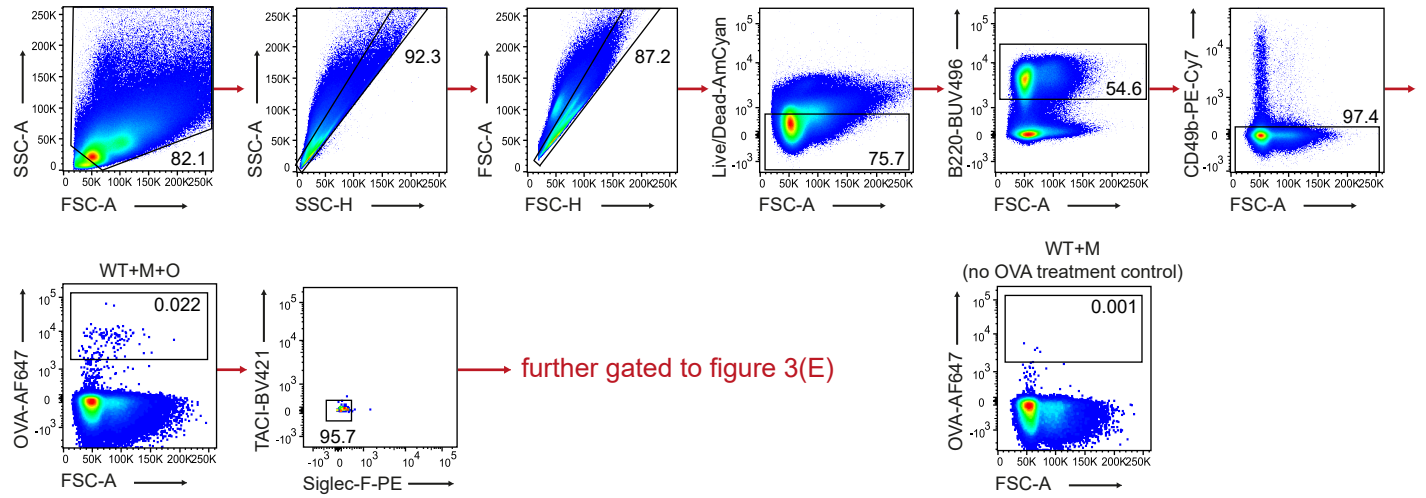

Supplementary figure 5\_Choi et al.

Supplement: Supplementary file 5 — Figure S5. Gating strategy for OVA‐specific B cells. [file ALL-81-220-s007.pdf]

Ear LN

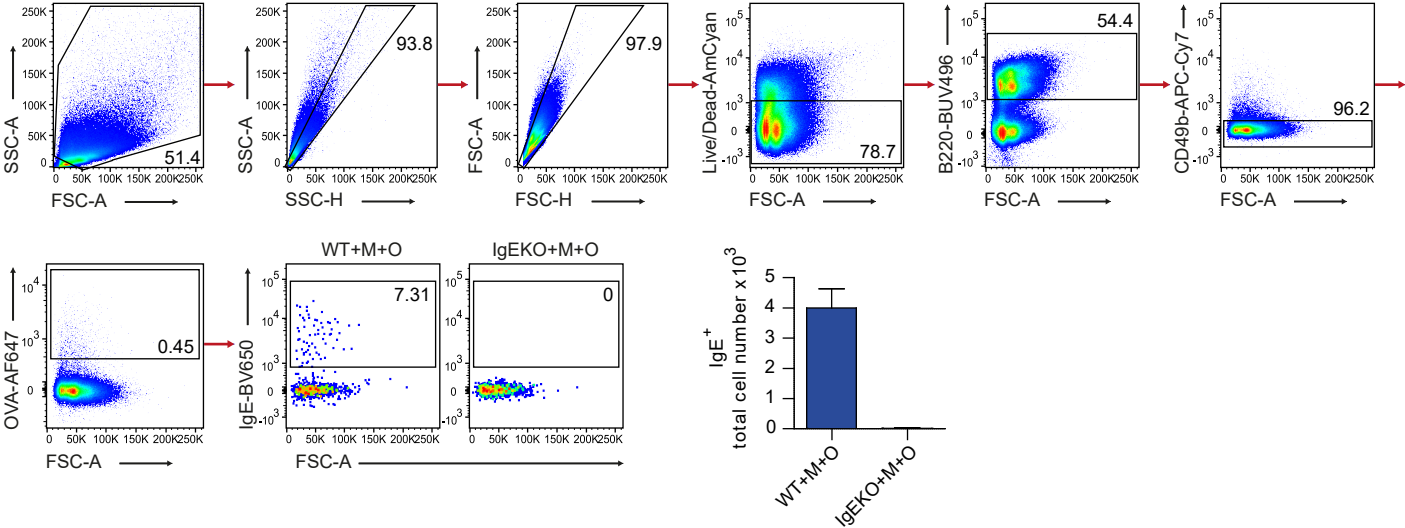

Lung

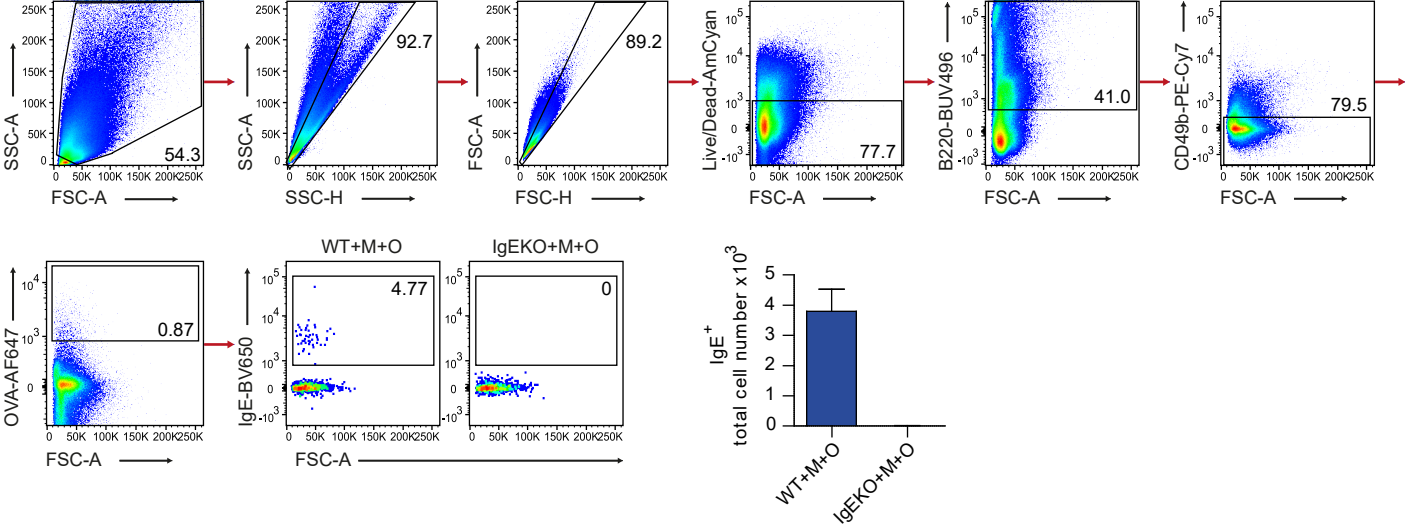

Supplementary figure 6\_Choi et al.

Supplement: Supplementary file 6 — Figure S6. Gating strategy and quantification of OVA‐specific IgE in WT and IgEKO mice. [file ALL-81-220-s001.pdf]
