# Supplementary material for: Basophils in Skin‐Mediated Sensitization Drive Subsequent Lung Inflammation in Airway‐Challenged Mice
Source: Allergy. 2025 Oct 11;81(1):220–31. doi: 10.1111/all.70093 (PMC12773652; doi:10.1111/all.70093)
Supplement: Supplementary file 2 — Figure S2. Quantification of basophils, CD4+ T, Th2, total IgE+ and IgG1+ GC B cells and PCs in ear LN; and OVA‐specific GC B cells and PCs in med LN. [file ALL-81-220-s002.pdf]

Ear LN (gated on living)

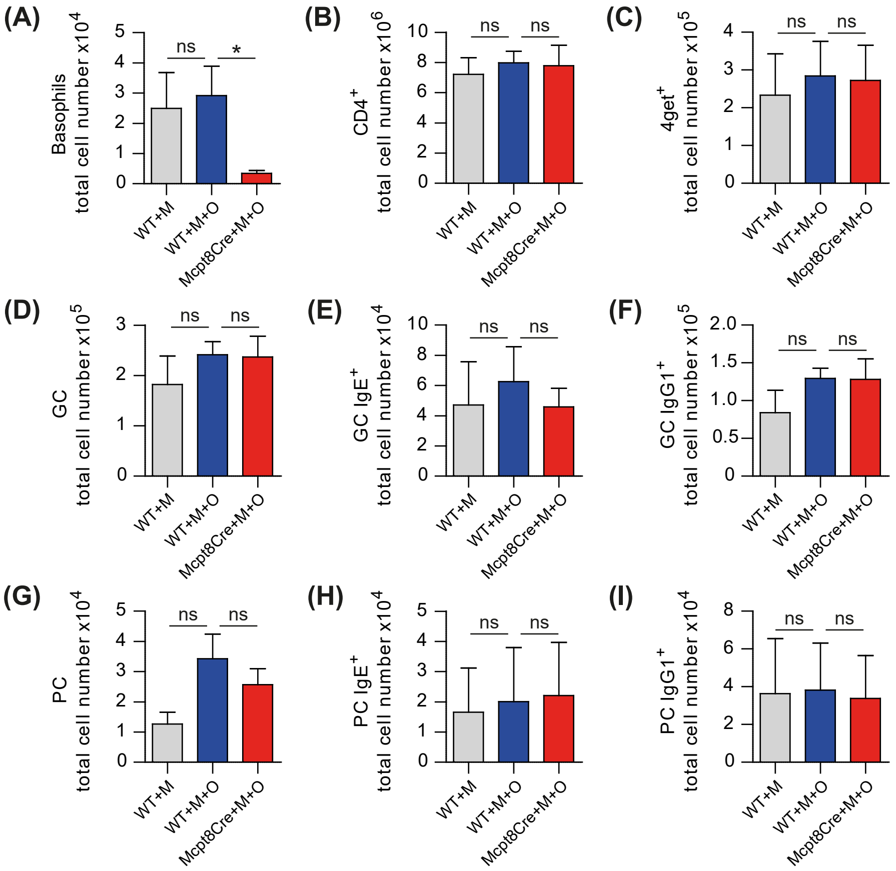

(J) Med LN (gated on living B220+CD38-CD95+GL7+)

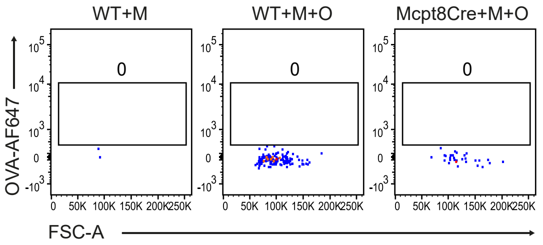

(K) Med LN (gated on living B220-TACI+CD138+)

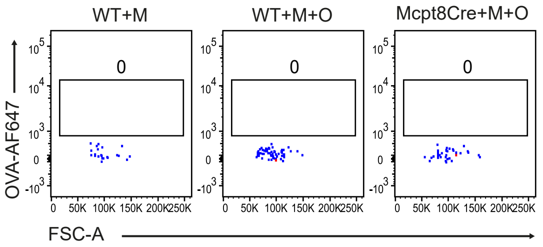

Supplementary figure 2_Choi et al.
