# Supplementary material for: Basophils in Skin‐Mediated Sensitization Drive Subsequent Lung Inflammation in Airway‐Challenged Mice
Source: Allergy. 2025 Oct 11;81(1):220–31. doi: 10.1111/all.70093 (PMC12773652; doi:10.1111/all.70093)
Supplement: Supplementary file 3 — Figure S3. Quantification and measurement of various control groups. [file ALL-81-220-s006.pdf]

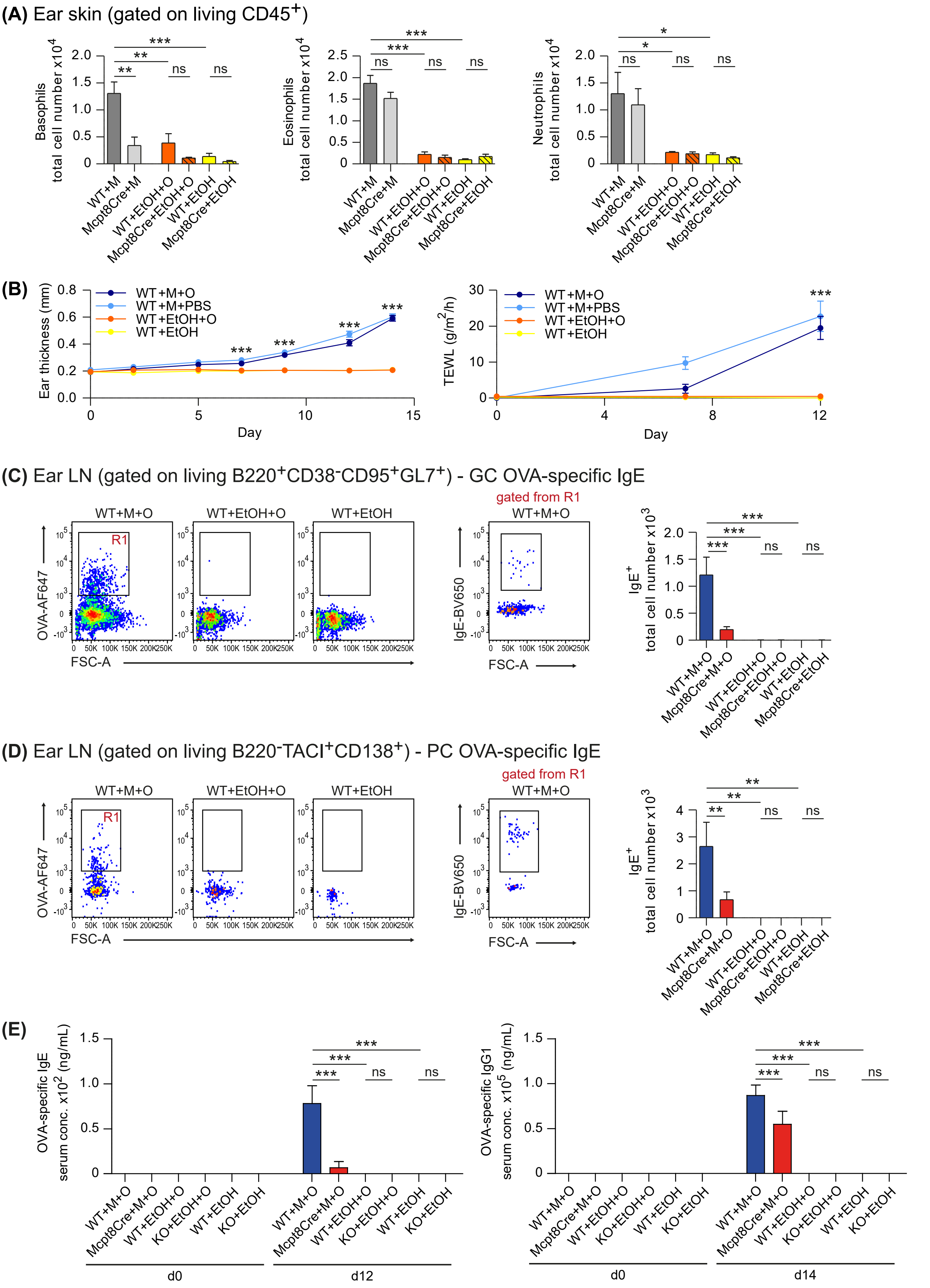

Supplementary figure 3_Choi et al.
