# Supplementary material for: Basophils in Skin‐Mediated Sensitization Drive Subsequent Lung Inflammation in Airway‐Challenged Mice
Source: Allergy. 2025 Oct 11;81(1):220–31. doi: 10.1111/all.70093 (PMC12773652; doi:10.1111/all.70093)
Supplement: Supplementary file 7 — Table S1. List of antibodies used for immunofluorescence staining. Table S2. List of primers used for qRT‐PCR analysis. [file ALL-81-220-s005.docx]

**Supplementary Information**

**Title**

Basophils in skin-mediated sensitization drive subsequent lung inflammation in airway-challenged mice

**Authors**

E Da Choi^1^, David Voehringer^1,2^ and Daniel Radtke^1^

^1^Department of Infection Biology, University Hospital Erlangen and Friedrich-Alexander University Erlangen-Nuremberg (FAU), Germany

^2^FAU Profile Center Immunomedicine (FAU I-MED), Germany

**Supplementary Figure 1.** Gating strategy for granulocyte subsets, GC B cells and PCs.

**Supplementary Figure 2.** Quantification of basophils, CD4^+^ T, Th_2_, total IgE^+^ and IgG1^+^ GC B cells and PCs in ear LN; and OVA-specific GC B cells and PCs in med LN.

**Supplementary Figure 3.** Quantification and measurement of various control groups.

**Supplementary Figure 4.** Concentration of total IgE and IgG1 in serum.

**Supplementary Figure 5.** Gating strategy for OVA-specific B cells.

**Supplementary Figure 6.** Gating strategy and quantification of OVA-specific IgE in WT and IgEKO mice.

**Supplementary Table 1.** List of antibodies used for immunofluorescence staining.

**Supplementary Table 2.** List of primers used for qRT-PCR analysis.

**Supplementary Figure Legends**

**Supplementary Figure 1. Gating strategy for granulocyte subsets, GC B cells and PCs.** (A) Exemplary gating strategy of basophils, eosinophils and neutrophils of WT ear skin treated with MC903. The bar graph represents total cell count of ear skin (both ears). (B) Exemplary gating strategy of OVA-specific total GC B cells and PCs from the ear-draining LN of OVA-sensitized and non-sensitized WT mice. WT = Mcpt8Cre- mice, Mcpt8Cre = basophil-deficient mice, M = MC903, O = OVA.

**Supplementary Figure 2. Quantification of basophils, CD4^+^ T, Th_2_, total IgE^+^ and IgG1^+^ GC B cells and PCs in ear LN and OVA-specific GC B cells and PCs in med LN.** Ear LNs of OVA-sensitized WT and Mcpt8Cre littermates were analyzed by flow cytometry. (A) Quantification of basophils. (B) Quantification of CD4^+^ T cells. (C) Quantification of 4get^+^ (Th_2_) cells. (D) Quantification of total GC cells, IgE^+^ (E) and IgG1^+^ (F). (G) Quantification of total PC cells, IgE^+^ (H) and IgG1^+^ (I). Med LNs of OVA-sensitized WT and Mcpt8Cre littermates were analyzed by flow cytometry (J-K) Flow cytometric representation of OVA-specific GC (J) or PC (K) cells. Data in (A-I) are pooled from three independent experiments with 6-7 mice per group. WT = Mcpt8Cre- mice, Mcpt8Cre = basophil-deficient mice, M = MC903, O = OVA. Bars and error bars represent means + SEM. **p* < 0.05; ***p* < 0.01; ****p* < 0.001. Two-way ANOVA (A-I).

**Supplementary Figure 3. Quantification and measurement of various control groups.** Both ear skin of WT and Mcpt8Cre mice were treated with various control solvents to exclude irritative effects of the solvent. (A) Quantification of basophils, eosinophils, and neutrophils in ear skin. (B) Ear thickness and TEWL measurement over time. (C-D) Flow cytometric representation and quantification of OVA-specific IgE^+^ total GC B cells (C), or PCs (D). (E) Serum concentration of OVA-specific IgE and IgG1 of OVA- and non-OVA-sensitized mice via the ear skin. Data in (A-E) are pooled data from two independent experiments with 4 mice per group, with one representative gating data (C-D). WT = Mcpt8Cre- mice, Mcpt8Cre = basophil-deficient mice, M = MC903, O = OVA, EtOH = ethanol, PBS = phosphate buffer saline. Bars and error bars represent mean + SEM. **p* < 0.05; ***p* < 0.01; ****p* < 0.001. One-way ANOVA (A, C-E) or two-way ANOVA (B).

**Supplementary Figure 4. Concentration of total IgE and IgG1 in serum.** Serum concentration of total IgE and IgG1 in mice OVA- or non-sensitized via the skin over time. Data are pooled from three independent experiments with 6-7 mice per group. WT = Mcpt8Cre- mice, Mcpt8Cre = basophil-deficient mice, M = MC903, O = OVA. Bars and error bars represent means + SEM. **p* < 0.05; ***p* < 0.01; ****p* < 0.001. Two-way ANOVA.

**Supplementary Figure 5. Gating strategy for OVA-specific B cells.** Exemplary gating strategy of OVA-specific B cells of OVA- and non-OVA-sensitized WT in lung and spleen. WT = Mcpt8Cre- mice, M = MC903, O = OVA.

**Supplementary Figure 6. Gating strategy and quantification of OVA-specific IgE in WT and IgEKO mice.** Exemplary gating strategy of OVA-specific IgE cells of OVA-sensitized WT and IgEKO mice in ear LN and lung. Data represents 5 mice per group. WT = Mcpt8Cre- mice, IgEKO = IgE-deficient mice, M = MC903, O = OVA.

**Supplementary Table 1. List of antibodies used for immunofluorescence staining.**

| **Antibody** | **Clone** | **Conjugate** | **Working dilution** | **Source** | **Identifier** |
| --- | --- | --- | --- | --- | --- |
| CD4 | RM4-5 | BV711 | 1:400 | BioLegend | 100550 |
| CD16/32 | 2.4G2 | Purified | 1:200 | Selleckchem | A2110 |
| CD38 | 90 | PerCP-Cy5.5 | 1:400 | BioLegend | 102722 |
| CD45R (B220) | RA3-6B2 | BUV496 | 1:200 | BD Bioscience | 621950 |
| CD49b | DX5 | APC-Cy7 | 1:100 | eBioscience^TM^ | 47-5971-82 |
|  | DX5 | PE-Cy7 | 1:100 | eBioscience^TM^ | 25-5971-82 |
| CD95 | Jo2 | BUV395 | 1:100 | BD Bioscience | 740254 |
| CD117 | 2B8 | PerCP-Cy5.5 | 1:100 | eBioscience^TM^ | 46-1171-82 |
| CD138 | 281-2 | BV785 | 1:200 | BioLegend | 142534 |
| CD267 (TACI) | 8F10 | BV421 | 1:100 | BD Bioscience | 742840 |
| IgE | R35-72 | BV650 | 1:300 | BD Bioscience | 564208 |
| IgG1 | A85-1 | BUV737 | 1:800 | BD Bioscience | 741733 |
| IgM | II/41 | APC-eFluor™ 780 | 1:200 | eBioscience^TM^ | 47-5790-82 |
|  | II/41 | PE-Cy7 | 1:100 | eBioscience^TM^ | 25-5790-82 |
| GL7 | GL7 | PE | 1:200 | BioLegend | 144608 |
| Siglec-F | E50-2440 | PE | 1:200 | BD Bioscience | 552126 |

| **Other staining reagents** | **Clone** | **Conjugate** | **Working dilution** | **Source** | **Identifier** |
| --- | --- | --- | --- | --- | --- |
| Fixable Viability Dye | - | eFluor^TM^ 506 | 1:800 | Invitrogen | 65-0866-14 |
| OVA | - | AF647 | 1:1000 | Thermo Fisher | O34784 |

**Supplementary Table 2. List of primers used for qRT-PCR analysis.**

| **Name** | **Oligonucleotides** | **Source** |
| --- | --- | --- |
| *Arg1* | F: CTCGCAAGCCAATGTACACG  R: GTATGACGTGAGAGACCACG | Microsynth AG |
| *Pdcd1lg2* | F: CTGCCGATACTGAACCTGAGC  R: GCGGTCAAAATCGCACTCC | Microsynth AG |
| *Ccl11* | F: TTCCATCTGTCTCCCTCCACCAT  R: CCTGGTCTTGAAGACTATGGCTTTCA | Microsynth AG |
| *Ccl17* | F: GAATGGCCCCTTTGAAGTAA  R: TGCTTCTGGGGACTTTTCTG | Microsynth AG |
| *Ccl24* | F: TCTTCCCCATAGATTCTGTGACCA  R: GTTTTTGTATGTGCCTCTGAACCC | Microsynth AG |
| *Col1a1* | F: CTGGCGGTTCAGGTCCAATGGG  R: GGGCTCTCCCTTAGGACCAGCA | Microsynth AG |
| *Fn1* | F: GCAAGCCTGAGCCTGAAGAGAC  R: CTTCTCAGCTATAGGTTTGCAGGTCC | Microsynth AG |
| *Il4* | F: ACTTGAGAGAGATCATCGGCA  R: AGCTCCATGAGAACACTAGAGTT | Microsynth AG |
| *Il13* | F: TGCTTTGTGTAGCTGAGCAG  R: GCAGTCCTGGCTCTTGCTTG | Microsynth AG |
| *Mmp12* | F: TTGCATTTGGAGCTCACGGAGACT  R: TCAAGGATGGGGGTTTCACTGGG | Microsynth AG |
| *Mmp13* | F: CACCAGAATCTATGATGGCACTGC  R: CATCGCCTGGACCATAAAGAAACT | Microsynth AG |
| *Muc5ac* | F: GGCTAACCTGTGGGCTCTGTGGTA  R: CACAAGCACGCACATAGGAGGACAG | Microsynth AG |
| *Retnla* | F: CTGCTGGGATGACTGCTAC  R: CTGGGTTCTCCACCTCTTC | Microsynth AG |
| *Retnlb* | F: AAGCCTACACTGTGTTTCCTTTT  R: GCTTCCTTGATCCTTTGATCCAC | Microsynth AG |
